# Molecular Mechanisms of PARP-1 Inhibitor 7-Methylguanine

**DOI:** 10.3390/ijms21062159

**Published:** 2020-03-20

**Authors:** Dmitry Nilov, Natalya Maluchenko, Tatyana Kurgina, Sergey Pushkarev, Alexandra Lys, Mikhail Kutuzov, Nadezhda Gerasimova, Alexey Feofanov, Vytas Švedas, Olga Lavrik, Vasily M. Studitsky

**Affiliations:** 1 Lomonosov Moscow State University, Belozersky Institute of Physicochemical Biology, Lenin Hills 1, bldg. 40, 119991 Moscow, Russia; vytas@belozersky.msu.ru; 2 Lomonosov Moscow State University, Biology Faculty, Lenin Hills 1, bldg. 12, 119992 Moscow, Russia; mal_nat@mail.ru (N.M.); lys-alex-bio-msu@yandex.ru (A.L.); shordome@gmail.com (N.G.); avfeofanov@yandex.ru (A.F.); 3 Institute of Chemical Biology and Fundamental Medicine, Siberian Branch of the Russian Academy of Sciences, Lavrentiev avenue 8, 630090 Novosibirsk, Russia; t.a.kurgina@gmail.com (T.K.); kutuzov.mm@mail.ru (M.K.); lavrik@niboch.nsc.ru (O.L.); 4 Novosibirsk State University, Pirogov str. 2, 630090 Novosibirsk, Russia; 5 Lomonosov Moscow State University, Faculty of Bioengineering and Bioinformatics, Lenin Hills 1, bldg. 73, 119991 Moscow, Russia; spush.bio@gmail.com; 6 Shemyakin-Ovchinnikov Institute of Bioorganic Chemistry, Russian Academy of Sciences, Miklukho-Maklaya str. 16/10, 117997 Moscow, Russia; 7 Fox Chase Cancer Center, Cottman Avenue 333, Philadelphia, PA 19111-2497, USA

**Keywords:** 7-methylguanine, poly(ADP-ribose) polymerase 1, inhibitor, nucleosome, trapping, docking, molecular dynamics, fluorescence anisotropy, spFRET microscopy

## Abstract

7-Methylguanine (7-MG), a natural compound that inhibits DNA repair enzyme poly(ADP-ribose) polymerase 1 (PARP-1), can be considered as a potential anticancer drug candidate. Here we describe a study of 7-MG inhibition mechanism using molecular dynamics, fluorescence anisotropy and single-particle Förster resonance energy transfer (spFRET) microscopy approaches to elucidate intermolecular interactions between 7-MG, PARP-1 and nucleosomal DNA. It is shown that 7-MG competes with substrate NAD^+^ and its binding in the PARP-1 active site is mediated by hydrogen bonds and nonpolar interactions with the Gly863, Ala898, Ser904, and Tyr907 residues. 7-MG promotes formation of the PARP-1–nucleosome complexes and suppresses DNA-dependent PARP-1 automodification. This results in nonproductive trapping of PARP-1 on nucleosomes and likely prevents the removal of genotoxic DNA lesions.

## 1. Introduction

Recently, various chemotherapy regimens including inhibitors of DNA repair enzyme poly(ADP-ribose) polymerase 1 (PARP-1), which selectively target BRCA-deficient tumors, have been extensively evaluated [1,2,3,4]. The underlying concept is that the cell death can be induced by simultaneous inhibition/inactivation of two key DNA repair molecules, PARP-1 and BRCA (breast cancer susceptibility protein), involved in different pathways of removing DNA lesions. PARP-1 binds to DNA breaks and synthesizes a signal polymer poly(ADP-ribose) (PAR) from NAD^+^ molecules to activate the excision repair proteins [5,6,7,8]. Upon PARP-1 inhibition, the number of double-strand DNA breaks is increased; however, they can still be removed using homologous recombination (i.e., through an alternative mechanism involving BRCA protein). If BRCA is deficient, genome instability reaches the critical level, resulting in cancer cell death [9,10,11,12]. A first-in-class PARP-1 inhibitor, a phthalazine derivative olaparib, was approved by FDA in 2014 and positioned as an innovative drug for the treatment of ovarian cancer in people with hereditary BRCA mutations [1,13,14]. Success of olaparib has inspired further studies of PARP inhibition, but also revealed serious side effects (in particular, hematological toxicity) that accompany the use of synthetic inhibitors [15,16,17,18,19]. The toxicity is likely related to the nonselective interaction with numerous NAD^+^-binding proteins (including other PARP family members, such as PARP-2) as well as to nonspecific effects on the organism characteristic for the synthetic molecules.

One way to reduce adverse effects of chemotherapy might be the use of natural PARP inhibitors instead of synthetic compounds. Although strong suppression of PARP seems to be inherently toxic due to an important role played by these proteins in the organism, attempts are continuing to find the proper balance between efficacy of natural inhibitors and their toxicity. For example, PARP inhibitors were identified among caffeine metabolites [20,21] and 2,5-diketopiperazines from chicken essence (a food supplement in Asian countries) [22]. Recently we have shown that a natural nitrogenous base, 7-methylguanine (7-MG), inhibits PARP-1 in vitro and accelerates apoptotic death of BRCA-deficient breast cancer cells induced by cisplatin and doxorubicin [23]. 7-MG has an attractive predicted profile of pharmacokinetics and toxicity and exerts no significant adverse effects on the organism in preliminary in vivo tests [24]. It contains a lactam group built in an aromatic scaffold (a common structural feature of effective PARP inhibitors [25,26]) and is expected to form substrate-specific interactions with the Gly863 and Tyr907 residues in the PARP-1 active site.

In this article, we present the results of a study of the 7-MG inhibition mechanism, which includes molecular modeling of the 7-MG binding to PARP-1, kinetics analysis of its ability to suppress the PARP-1-catalysed synthesis of PAR, and microscopy analysis of interaction between 7-MG, PARP-1 and nucleosomal DNA.

## 2. Results

### 2.1. MD Modeling

**Figure 1:**
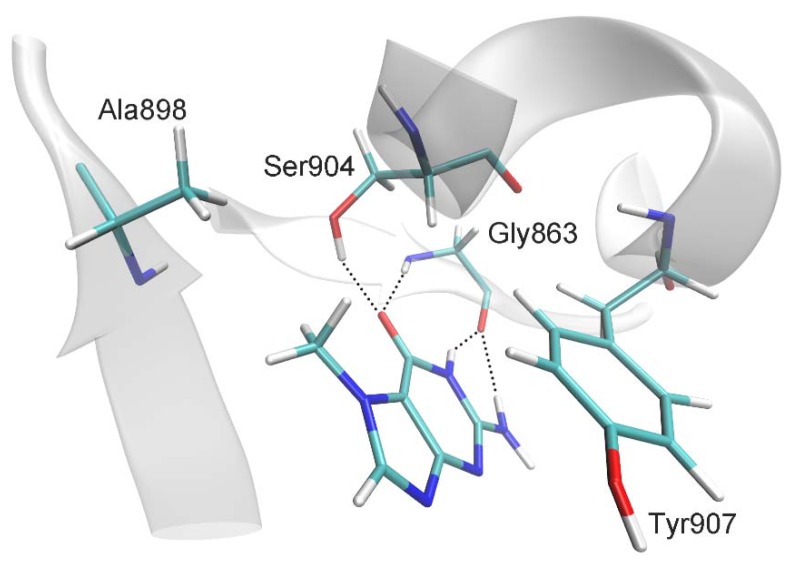
Interactions of 7-MG molecule in the PARP-1 active site revealed by MD simulation: hydrogen bonds with Gly863 and Ser904, *π*-stacking of purine rings with Tyr907, and hydrophobic contact between the 7-MG methyl group and Ala898.

Molecular dynamics (MD) modeling of the complex of multidomain human PARP-1 with DNA and 7-MG (Figure S1) has been performed for the first time. 7-MG was docked into its putative binding region (binding site of the NAD^+^ nicotinamide group), and the obtained complex was subjected to MD simulation in explicit solvent. The system contained 703 amino acid residues, 52 nucleotides, 2 Zn^2+^ ions, 45 Na^+^ ions, and 73,302 water molecules (232,862 atoms). Analysis of the 20-ns equilibrium simulation trajectory revealed the following important intermolecular interactions. The lactam group and 2-amino group of 7-MG form hydrogen bonds with the Gly863 residue (Figure 1 and Table 1). 7-Methyl group forms a hydrophobic contact with the Ala898 side chain, and purine rings stack with the Tyr907 side chain. An additional hydrogen bond is formed between the 7-MG lactam group and the Ser904 side chain. This interaction is characterized by an increased mean distance between atoms (Table 1) because Ser904 periodically forms an alternative hydrogen bond with the Trp861 backbone and thus can be observed in two possible conformations (Figure S2a,b). However, the hydrogen bond of Ser904 with 7-MG is more prevalent, with the occupancy of about 60% (Figure S2c). As demonstrated in Figures S3 and S4, the position of 7-MG and its hydrogen bonds with protein were stable during the simulation. Noticeably, we did not observe significant changes in the PARP-1 multidomain organization upon 7-MG binding (compared with the PARP-1–DNA crystal structure); thus the inhibitor apparently does not affect the interaction between PARP-1 and DNA in the case of double-stranded oligonucleotides.

### 2.2. Fluorescence Anisotropy Analysis

PARP-1 inhibition by 7-MG was studied using analysis of the fluorescence anisotropy of PARP-1 complexes with a labeled double-stranded oligonucleotide. Upon PARP-1 binding to DNA, the anisotropy level is increased due to a decrease in the fluorophore mobility. The addition of the NAD^+^ substrate enables synthesis of negatively charged PAR and automodification of the enzyme, leading to the dissociation of the PARP-1–DNA complexes accompanied by a decrease in anisotropy. The observed dissociation rate is proportional to the PARP-1-catalyzed reaction rate [27].

**Figure 2:**
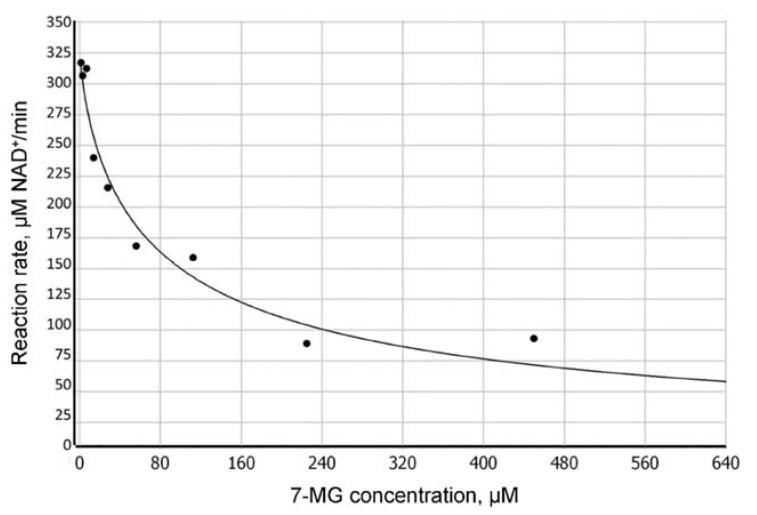
Dependence of the PARP-1-catalyzed reaction rate on the concentration of 7-MG inhibitor determined by fluorescence anisotropy (100 μM NAD^+^ concentration).

**Figure 3:**
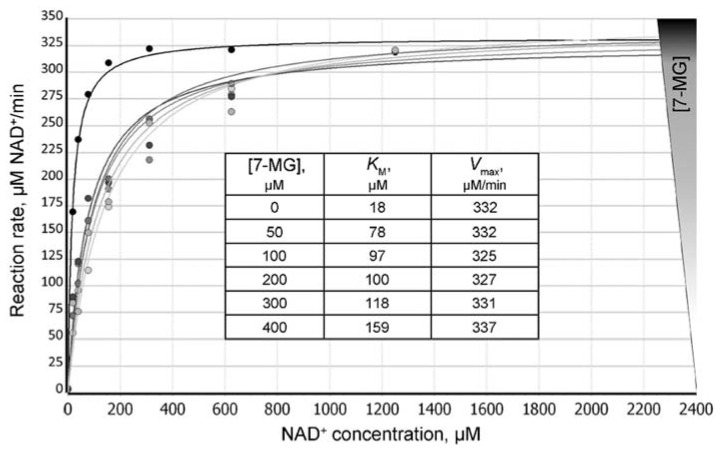
Dependence of the PARP-1-catalyzed reaction rate on the NAD^+^ concentration at different concentrations of 7-MG added to the reaction mixture. Insert: calculated *K*_M_^app^ values increase with increasing 7-MG concentrations, thus demonstrating the competitive inhibition mechanism.

We have found that 7-MG does not affect the oligonucleotide structure by itself and does not significantly interfere with the binding of PARP-1 to DNA (Figure S5), but inhibits the dissociation of the PARP-1–DNA complex (due to suppression of PARP-1 catalytic activity). Figure 2 shows a typical plot of the reaction rate as a function of 7-MG concentration. The absolute IC_50_ [28] was found to be 162 ± 4 μM at 100 μM NAD^+^ concentration. To determine the type of inhibition, we calculated apparent *K*_M_ and *V*_max_ values at various 7-MG concentrations (Figure 3). The 7-MG addition altered only the *K*_M_^app^ leaving the *V*_max_ value the same, which displays the competitive enzyme inhibition; the corresponding *K*_i_ value was found to be 61 ± 9 μM.

### 2.3. spFRET Analysis

To model the inhibitor activity in the chromatin environment, we have studied effects of 7-MG on the PARP-1 complexes with fluorescently labeled nucleosomes P147 and P167 using single-particle Förster resonance energy transfer (spFRET) microscopy. Cy3 and Cy5 labels were introduced in the neighboring DNA gyres and served to probe DNA conformation near the entrance of DNA into the nucleosome by measuring FRET efficiency between the labels.

**Figure 4:**
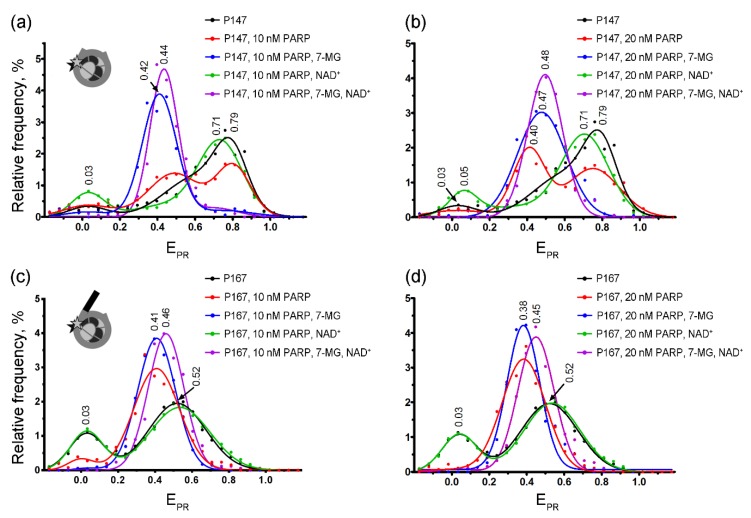
spFRET studies of 7-MG effects at the nucleosome level. Typical frequency distributions of P147 (**a**,**b**) and P167 (**c**,**d**) nucleosomes by E_PR_ in different mixtures are shown. Conditions: 1 nM nucleosomes, 10 nM or 20 nM PARP-1, 100 μM NAD^+^, 450 μM 7-MG. Inserts: schemes of nucleosome structure and positions of fluorescent labels (asterisks).

spFRET microscopy revealed the presence of two subpopulations for both P147 and P167 nucleosomes in solution, which differed in FRET efficiency (presented as the proximity ratio E_PR_ [29] in Figure 4). These subpopulations were observed in the calculated E_PR_ profiles of nucleosomes (i.e., frequency distributions of nucleosomes by E_PR_) as two peaks. In agreement with our previous work [30], the major peaks (E_PR_ ≈ 0.79 for P147 and E_PR_ ≈ 0.52 for P167) can be assigned to nucleosome subpopulations with tightly wrapped nucleosomal DNA, while the minor peaks (E_PR_ ≈ 0.03 for P147 and P167) can be related to nucleosomes with partially unwrapped DNA and/or free DNA. Differences in the E_PR_ profiles of P147 and P167 nucleosomes are related with the presence of the 20-bp linker DNA arm in the P167 that seems to affect both wrapping of DNA on the surface of the histone octamer and DNA “breathing” (temporal spontaneous partial DNA unwrapping) near the entrance of DNA into nucleosome.

The formation of the complexes between nucleosomes (P147 or P167) and PARP-1 resulted in the appearance of a new peak characterized by E_PR_ ≈ 0.38–0.41 in the E_PR_ profiles (Figure 4). Relative intensity of this peak indicates that only a part of P147 nucleosomes formed complexes at the studied PARP-1 concentrations (10–20 nM), and this subpopulation was increased after the increase in the PARP-1 concentration. In contrast, nearly all P167 nucleosomes formed the complexes even at 10 nM concentration of PARP-1, indicating higher affinity of PARP-1 to nucleosomes with a linker DNA arm. The PARP-1-induced shift of the main E_PR_ peak to lower values is related to structural changes in nucleosomal DNA, which are accompanied by an increase in the distance between neighboring DNA gyres in the region of Cy3 and Cy5 label position, i.e., at the H2A–H2B interface (+13 bp position) and the H4–H2B interface (+91 bp position) [30]. Although 7-MG alone did not affect the nucleosome structure (Figure S6), its addition to P147 together with PARP-1 resulted in a considerable increase of the fraction of nucleosomes with the E_PR_ distribution having maximum at 0.42–0.47 (Figure 4a,b), thus indicating that (i) 7-MG is involved in the formation of complexes of nucleosomes with PARP-1, (ii) 7-MG only weakly disturbs the structure of nucleosomal DNA in the PARP-1–nucleosome complexes, (iii) 7-MG facilitates PARP-1 binding to nucleosomes. Similarly, spFRET analysis demonstrated the ability of 7-MG to promote PARP-1 binding to P167 nucleosome; however, the effect was less pronounced than in the case of P147 nucleosome (Figure 4c,d).

Incubation of PARP-1–nucleosome complexes with NAD^+^ resulted in the changes of the E_PR_ profiles characterized by disappearance of the peak at E_PR_ ≈ 0.38–0.41 (a signature of nucleosome-PARP-1 complexes) and appearance of the peak in the region of E_PR_ ≈ 0.52 (for P167) or E_PR_ ≈ 0.71 (for P147), which was characteristic for intact nucleosomes (Figure 4). The data indicate that, as expected, PARP-1 automodification resulted in its dissociation from nucleosomal DNA and almost complete recovery of the intact nucleosome structure. The presence of 7-MG has blocked the described changes in the E_PR_ profiles of enzyme–nucleosome complexes induced by addition of NAD^+^: only a small shift of the peak at E_PR_ ≈ 0.38–0.47 was observed without appearance of the subpopulation of free nucleosomes (no peaks with a maximum at E_PR_ ≈ 0.52 for P167 or E_PR_ ≈ 0.71–0.79 for P147). This effect of 7-MG is likely related to inhibition of PARP-1 enzymatic activity that prevents the automodification of PARP-1 and its dissociation from the nucleosomes.

## 3. Discussion

In our earlier study, we have modeled a complex of PARP-1 with 7-MG [23]; however, the preliminary data were obtained using an isolated catalytic domain of chicken PARP-1 and might be incomplete. Here we report the MD model of human multidomain PARP-1 complex with DNA fragment and 7-MG that most accurately describes interactions between the inhibitor and PARP-1 bound to a DNA double-strand break. In the refined model, 7-MG formed polar and hydrophobic interactions, in particular hydrogen bonds with Gly863, similar to the NAD^+^ substrate and known effective PARP-1 inhibitors [15,25,26]. One important difference from chicken PARP-1 model was the formation of an additional hydrogen bond between 7-MG and Ser904 which apparently contributes to the binding of the inhibitor with human PARP-1. Revealed interactions of 7-MG in the PARP-1 active site and the stability of the enzyme–inhibitor complex during MD simulation suggest that 7-MG occupies the binding site of the NAD^+^ nicotinamide group. Kinetics analysis of the ability of 7-MG to suppress PAR synthesis was performed using a recently developed fluorescent method for the real-time measurement of PARP-1 activity [27]; it corroborated conclusions of MD modeling that 7-MG is a competitive PARP-1 inhibitor.

To confirm the inhibitory properties at a more complex level, 7-MG effects have been investigated using spFRET microscopy, an advanced technique allowing analysis of structurally different subpopulations of nucleosomes in heterogeneous samples [31,32,33]. Mononucleosomes used in our study represented a convenient model of DNA double-strand break in the chromatin environment. Although 7-MG exerted no significant effect on the PARP-1 interaction with isolated double-stranded oligonucleotides (as shown using MD modeling and fluorescence anisotropy experiments), spFRET data showed that it promotes PARP-1 binding to nucleosomes. It seems that the inhibitor stabilizes the interaction between PARP-1 and nucleosomal DNA. We propose that the relative orientation of PARP-1 domains undergoes changes to fit the nucleosome structure, and bound 7-MG stabilizes the new PARP-1 conformation. The formed enzyme–nucleosome complexes are nonproductive because of the concomitant 7-MG inhibition of PARP-1 catalytic activity. PARP-1 molecules fail to regulate their dissociation from DNA via PAR synthesis and get trapped on DNA. The trapped PARP-1 complexes are considered to be even more deleterious for cancer cells than unrepaired DNA strand breaks, because PARP-1 protein tightly bound to DNA interferes with transcription, replication, and DNA repair [34,35,36]. Noticeably, the most effective PARP-1 inhibitors, including olaparib, display the strong ability to trap PARP [37,38].

In conclusion, the molecular mechanisms of a promising anticancer compound, 7-MG, can be outlined as follows. (1) 7-MG forms substrate-specific interactions in the PARP-1 active site and inhibits the synthesis of PAR, a signal polymer that induces the reorganization of chromatin structure and recruits DNA repair proteins to eliminate the damage. (2) 7-MG inhibits the dissociation of PARP-1 from the DNA damage site in the context of nucleosome and likely prevents further steps in DNA repair, as well as DNA replication and transcription, inducing cancer cell death. Despite the fact that 7-MG is a weaker inhibitor compared to some synthetic PARP-1 inhibitors, we believe that this natural compound has more favorable profile of pharmacokinetics and toxicity, and therefore can be considered as a promising new component of chemotherapy.

## 4. Materials and Methods

### 4.1. Molecular Modeling

The molecular model of human PARP-1 bound to DNA was constructed on the basis of the 4dqy crystal structure (chains A, B, C, M, and N) [39,40]. The coordinates of the missing loop 576–583 in the WGR domain were transferred from the 2cr9 structure. The coordinates of the loop 645–661 between the WGR and catalytic domains were predicted with the Modeller 9.20 program [41]. Next, the protein and DNA structure was optimized with AmberTools 15 and Amber 14 [42,43], according to the following protocol. Hydrogen atoms were added to the structure considering ionization of amino acid residues, and then it was solvated by 12 Å-thick layer of TIP3P water; sodium ions were added to neutralize the negative net charge. The two-stage energy minimization was then performed to relax the solvated system. At the first stage (2500 steps of the steepest descent algorithm + 2500 steps of the conjugate gradient algorithm), the DNA coordinates of the protein and DNA were kept fixed by the positional restraints of 2 kcal/(mol∙Å^2^) on heavy atoms. The second minimization stage (5000 steepest descent steps + 5000 conjugate gradient steps) was carried out without restraints.

To obtain the starting model of the PARP-1–DNA–7-MG complex, water molecules and ions were removed from the optimized PARP-1–DNA complex and then it was subjected to molecular docking with Lead Finder 1.1.16 [44,45]. The 7-MG molecule was docked into the active site of the PARP-1 catalytic domain using the genetic algorithm. The obtained ternary complex was re-solvated and re-optimized using the protocol described above, and subsequently studied through molecular dynamics simulation. The system was heated up from 0 to 300 K with positional restraints of 1 kcal/(mol∙Å^2^) on the protein, DNA, and 7-MG atoms (250 ps, constant volume) and equilibrated at 300 K (500 ps, constant pressure). Lastly, a 20 ns trajectory of equilibrium simulation was calculated and analyzed. Control data for energy minimization and MD simulation are provided in Table S1. The *ff14SB* force field [46] was used to describe the protein and DNA with molecular mechanics, and recently developed parameters [23] were used to describe the 7-MG molecule. VMD 1.9.2 was used for the visualization of structures [47].

### 4.2. Fluorescence Anisotropy Assay

PARP-1 protein was obtained from the insect cells using baculovirus expression system. A suspension of Hi5 cells in serum-free medium (2∙10^6^ cells/mL) was infected with baculovirus (10 pfu/mL) containing cDNA of PARP-1, a kind gift of V. Schreiber (Strasbourg, France). Insect cells were then collected by centrifugation during 10 min at 1000× *g*. The purification of PARP-1 was performed according to the earlier described protocol [48]. The fluorescein-labeled DNA duplex used in kinetics experiments is provided in Table S2.

Real-time measurements of the PARP-1 activity were based on recently developed fluorescent method [27]. The reaction mixture contained a buffer (50 mM Tris-HCl, pH 8.0, 50 mM NaCl, 1 mM DTT, 5 mM MgCl_2_), 200 nM PARP-1, 100 nM DNA, and 7-MG (0–450 μM). The reaction was started by adding NAD^+^ (100 μM). Fluorescence anisotropy measurements were performed at 25 °C using the CLARIOstar multifunctional microplate reader (BMG LABTECH, Ortenberg, Germany). The fluorescent probes were excited at 495 nm, and the fluorescence intensity was detected at 520 nm. To determine the IC_50_ value, the experiment was done in triplicate. To determine the type of enzyme inhibition, reaction mixtures containing the buffer, 7-MG (0–400 μM) and NAD^+^ (0–2500 μM) were used, and the reaction was started by adding a mixture of PARP-1 (200 nM) and DNA (100 nM).

Anisotropy was calculated using formula:

(1) $$A=\frac{\left(I_{\|}-I_{⊥}\right)}{\left(I_{\|}+2I_{⊥}\right)}$$

where I_‖_—fluorescence intensity parallel to the plane-polarized exited light, I_⊥_—fluorescence intensity perpendicular to the light.

### 4.3. spFRET Microscopy

Mononucleosomes were assembled using 147 or 167 bp DNA fragments containing strong nucleosome positioning sequence 603 (147 bp-long) [49,50]. Nucleosomes P147 contained only the 603 sequence, while P167 included an additional 20-bp linker. Cy3 and Cy5 fluorophore labels (Biotech Industry Ltd., Moscow, Russia) were introduced at +13 and +91 bp (from the beginning of the 603 sequence) to enable spFRET-microscopy detection of changes in the nucleosome structure. Label positions were selected in the neighboring DNA gyres based on the nucleosome crystal structure [51,52]. Sequences of DNA template and fluorescently labeled oligonucleotide primers are shown in Table S3. Nucleosomes were assembled with donor chromatin (lacking H1 histone) from chicken erythrocytes [53,54] using salt dialysis against buffers (10 mM Tris pH 7.5, 0.2 mM EDTA, 0.1% NP-40, 5 mM 2-mercaptoethanol) with decreasing concentrations of NaCl (1, 0.5, 0.2, 0.01 M ). The nucleosome assembly was controlled by a native polyacrylamide gel (4%) electrophoresis. Fluorescently-labeled nucleosomes (1 nM) were incubated with PARP-1 for 20 min in a buffer containing 50 mM Tris-HCl pH 8.0, 40 mM NaCl, 1mM DTT at 25 °C in siliconized tubes. To activate PARP-1, NAD^+^ was added to final concentration of 100 μM and the mixture was incubated for 15 min. In experiments with 7-MG, PARP-1 was preincubated with 450 μM of the inhibitor for 15 min and mixed with the nucleosomes.

Fluorescence of single nucleosomes and their complexes was measured during their free diffusion through the focus of a laser beam (wavelength of 514.5 nm) with the LSM 710 ConfoCor 3 confocal microscope (Zeiss, Oberkochen, Germany) as described elsewhere [32]. To characterize the distance between Cy3 (donor) and Cy5 (acceptor) dyes in neighboring gyres, a proximity ratio E_PR_ was calculated for each nucleosome:

(2) $$E_{PR}=\frac{\left(I_{Aa}-\alpha \times I_{Dd}\right)}{\left[I_{Aa}+\left(1-\alpha \right)\times I_{Dd}\right]}$$

where *I_Aa_* indicates the fluorescent intensity of the Cy5 label (Cy5 channel), *I_Dd_* indicates intensity of the Cy3 label (Cy3 channel) and *α* is a coefficient of spectral cross-talk calculated as:

(3) $$\alpha =\frac{I_{Da}}{I_{Dd}}$$

where *I_Da_* is a fluorescent intensity of the Cy3 in the Cy5 channel. Data collected from at least 2000 individual particles have been used to plot a relative frequency distribution of nucleosomes by E_PR_.

## Figures and Tables

**Table 1:** Distances and angles describing 7-MG position in the PARP-1 active site determined by the 20-ns MD simulation. Mean values are presented with standard deviations.

**Distance, Å**	
7-MG:CO:O ··· Gly863:H	2.0 ± 0.2
7-MG:CO:O ··· Ser904:OG:HG	2.5 ± 0.7
7-MG:NH:H ··· Gly863:O	1.9 ± 0.1
7-MG:NH_2_:H ··· Gly863:O	2.4 ± 0.3
7-MG:CH_3_:C ··· Ala898:CB	4.0 ± 0.4
*C*(7-MG fused rings) ··· *C*(Tyr907 benzene ring) ^1^	3.6 ± 0.2
**Angle, deg**	
7-MG:CO:O ··· Gly863:H ··· Gly863:N	162 ± 11
7-MG:CO:O ··· Ser904:OG:HG ··· Ser904:OG	134 ± 34
7-MG:NH:N ··· 7-MG:NH:H ··· Gly863:O	153 ± 11
7-MG:NH_2_:N ··· 7-MG:NH_2_:H ··· Gly863:O	138 ± 10

^1^ Distance between the geometric center of 7-MG fused rings and the center of the Tyr907 benzene ring.

## Supplementary Materials

Supplementary materials can be found at https://www.mdpi.com/1422-0067/21/6/2159/s1.

## Abbreviations

PARP-1	poly(ADP-ribose)polymerase 1
PAR	poly(ADP-ribose)
7-MG	7-methylguanine
MD	molecular dynamics
spFRET	Single-particle Förster resonance energy transfer
