# Supplementary material for: Molecular Mechanisms of PARP-1 Inhibitor 7-Methylguanine
Source: Int J Mol Sci. 2020 Mar 20;21(6):2159. doi: 10.3390/ijms21062159 (PMC7139824; doi:10.3390/ijms21062159)
Supplement: Supplementary file 1 [file ijms-21-02159-s001.pdf]

Supplementary Materials

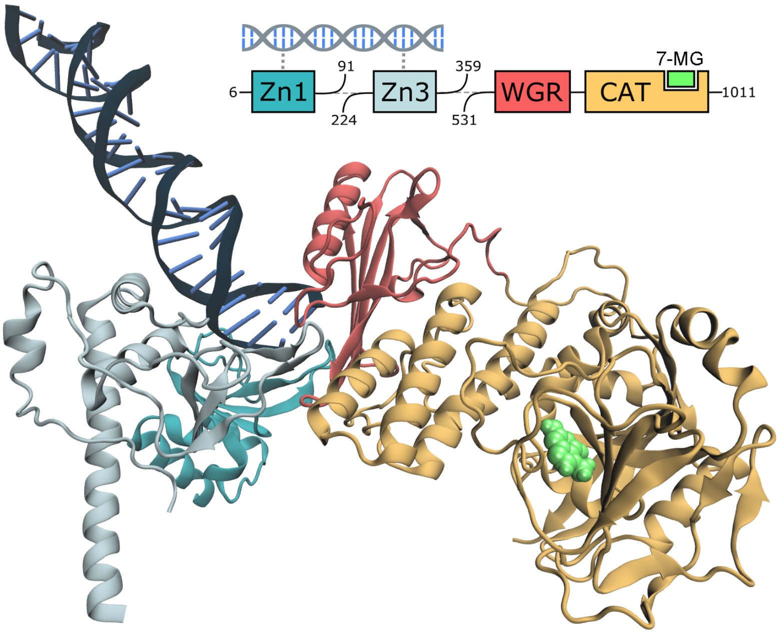

Figure S1. Overall view of the modeled PARP-1 complex with DNA and 7-MG inhibitor (shown in green).

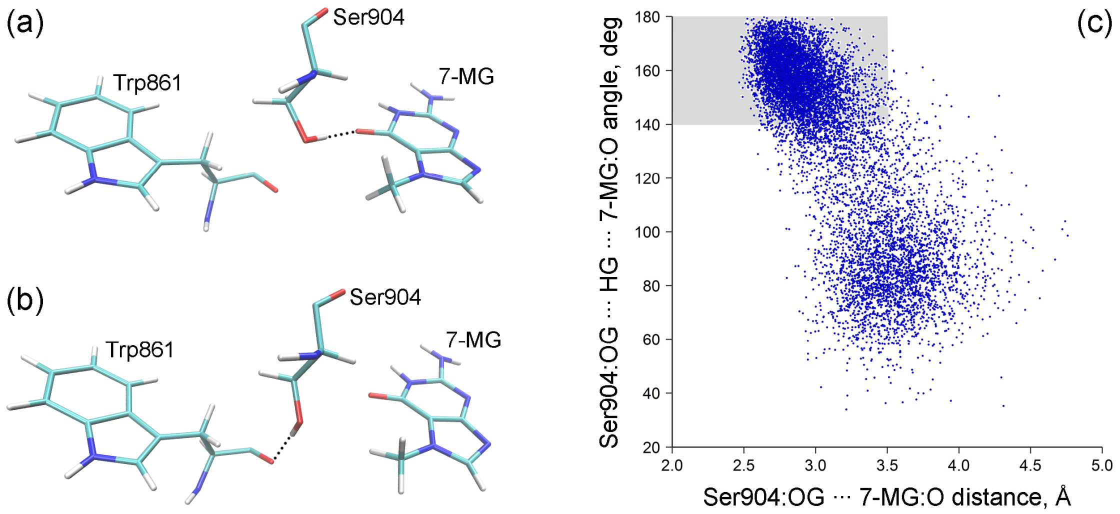

Figure S2. Two possible conformations of the Ser904 residue in the PARP-1 active site. (**a**) Conformation providing a hydrogen bond with the 7-MG lactam group; (**b**) Conformation providing a hydrogen bond with the Trp861 backbone; (**c**) Distribution of Ser904:OG ··· 7-MG:O distance and Ser904:OG ··· HG ··· 7-MG:O angle over the 20-ns MD trajectory. The area where the Ser904 hydroxyl group forms a hydrogen bond with 7-MG is shown in gray.

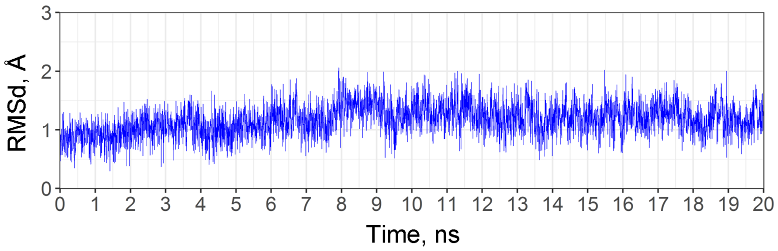

Figure S3. Mass-weighted RMS deviation of 7-MG during the 20-ns PARP-1 simulation. Trajectory frames were superimposed onto the final structure of the equilibration stage by fitting the backbone atoms of the PARP-1 catalytic domain, and then the 7-MG RMSd was calculated.

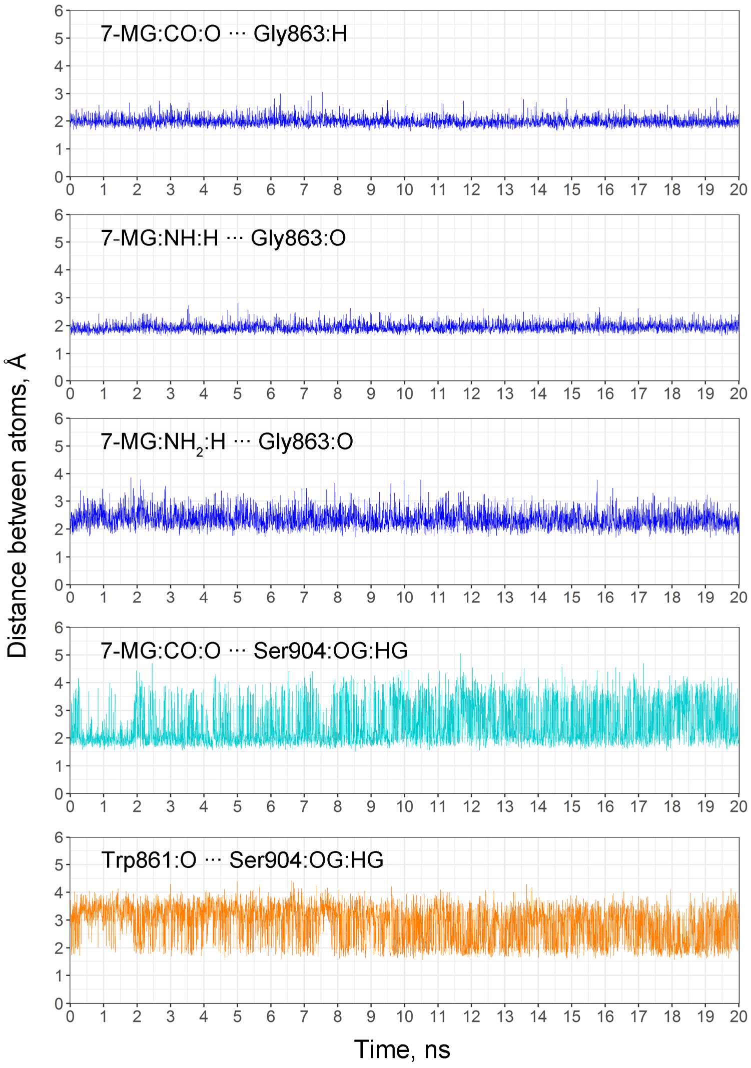

Figure S4. H-bond distances in the PARP-1 active site mediated by residues Gly863, Ser904, and Trp861 during the 20-ns MD simulation.

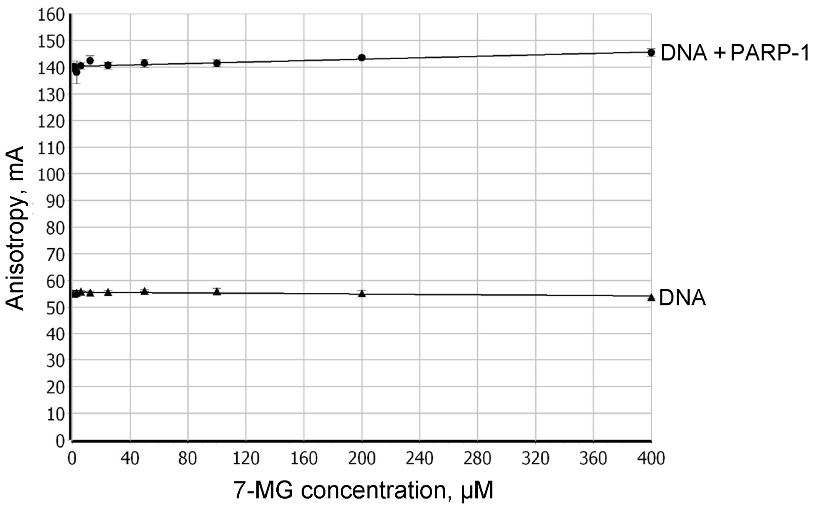

Figure S5. 7-MG does not affect significantly the DNA and PARP-1–DNA complex structures in fluorescence anisotropy experiments. Changes in the anisotropy level of the PARP-1–DNA complex induced by 7-MG are within 5% of anisotropy increase upon the complex formation and are thus very subtle.

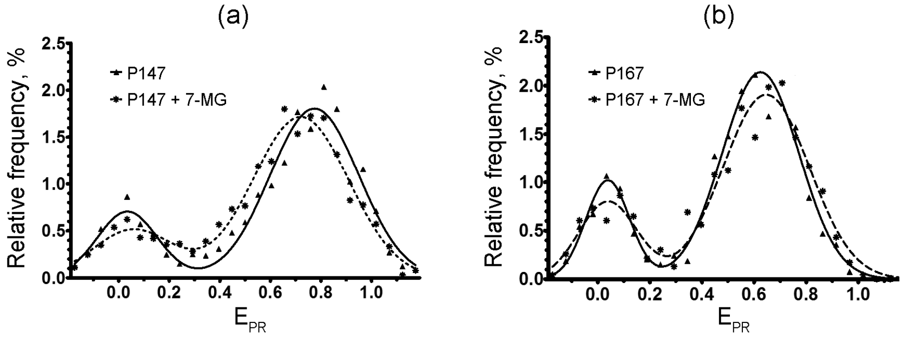

Figure S6. 7-MG does not affect the nucleosome structure. Relative frequency distributions of P147 (**a**) and P167 (**b**) nucleosomes (1 nM) by E_{PR} in the absence and presence of 7-MG (450 μM).

Table S1. Control data used for energy minimization and MD simulation of the PARP-1–DNA–7-MG complex. For more information see the Amber Users' Manual.

Energy minimization, stage 1	Energy minimization, stage 2
&cntrl<br>imin=1, maxcyc=5000, ncyc=2500,<br>cut=10.0, ntb=1,<br>ntc=1, ntf=1,<br>ntpr=10,<br>ntr=1,<br>restraintmask=':1-758 & !@H=',<br>restraint_wt=2.0<br>/	&cntrl<br>imin=1, maxcyc=10000, ncyc=5000,<br>cut=10.0, ntb=1,<br>ntc=1, ntf=1,<br>ntpr=10<br>/
**MD simulation, heating**	**MD simulation, equilibration**
&cntrl<br>imin=0, irest=0, ntx=1,<br>nstlim=125000, dt=0.002,<br>ntc=2, ntf=2,<br>cut=10.0, ntb=1,<br>ntpr=500, ntwx=500,<br>ntt=3, gamma_ln=2.0,<br>tempi=0.0, temp0=300.0,<br>ntr=1, restraintmask=':1-758',<br>restraint_wt=1.0,<br>nmropt=1<br>/<br>&wt TYPE='TEMP0',<br>istep1=0, istep2=125000,<br>value1=0.1, value2=300.0 /<br>&wt TYPE='END' /	&cntrl<br>imin=0, irest=1, ntx=5,<br>nstlim=250000,<br>*(to run 20 ns simulation, set nstlim=10000000)*<br>dt=0.002,<br>ntc=2, ntf=2,<br>cut=10.0, ntb=2, ntp=1, taup=2.0,<br>ntpr=1000, ntwx=1000, ntwr=50000,<br>ntt=3, gamma_ln=2.0,<br>temp0=300.0<br>/

Table S2. Oligonucleotide sequences (Biosset, Russia) and corresponding DNA duplex used in fluorescence anisotropy experiments. DNA duplex contains a nick (marked "|") and 6-carboxyfluorescein (FAM). To obtain the duplex, oligonucleotides were annealed at equimolar ratio (N1 was the matrix strand).

Oligonucleotide sequences
N1 5′-GGAAGACCCTGACGTTCCCAACTTTATCGCC-FAM-3′<br>N2 5′-GGCGATAAAGTTGGG-3′<br>N3 5′-AACGTCAGGGTCTTCC-3′
**DNA duplex**
5′-GGAAGACCCTGACGTTCCCAACTTTATCGCC-FAM-3′<br>3′-CCTTCTGGGACTGCAA|GGGTTGAAATAGCGG-5′

Table S3. Sequences of DNA template s603-42 and fluorescently labeled oligonucleotide primers used to assemble the P147 and P167 nucleosomes. PCR was performed using Evrogen (Russia) reagents. Reaction products were purified by Cleanup Standard Kit (Evrogen, Russia) following the manufacturer's protocol.

**Template sequence s603-42**
5′CCCGGTTCGCGCGCCCGCCTTCCGTGTGTTGTCGTCTCTCGAGCTTCTAAGTACGCTTAGC GCACGGTAGAGCGCAATCCAAGGCTAACCACCGTGCATCGATGTTGAAAGAGGCCCTCC GTCCTTATTACTTCAAGTCCCTGGGGT3′
**P147 nucleosomes**
Forward<br>5′CCCGGTTCGCGCT#CCCGCCTTCCGTGTGTTGTCGTCTCTCGG3′ (T# refers to a nucleotide labeled with Cy3)<br>Reverse<br>5′ACCCCAGGGACTTGAAGTAATAAGGACGGAGGGCCTCTTTCAACATCGATGCACGGT*G GTTAG3′ (T* refers to a nucleotide labeled with Cy5)
**P167 nucleosomes**
Forward<br>5′CAAGCGACACCGGCACTGGGCCCGGTTCGCGCT#CCCGCCTTCCGTGTGTTGTCGTCTCTC GGGCGT3′ (T# refers to a nucleotide labeled with Cy3)<br>Reverse<br>5′GAACCATGATGGGCACTGGGTACCCCAGGGACTTGAAGTAATAAGGACGGAGGGCCTC TTTCAACATCGATGCACGGT*GGTTAG3′ (T* refers to a nucleotide labeled with Cy5)
